# Implementing an interprofessional narrative medicine program in academic clinics: Feasibility and program evaluation

**DOI:** 10.1007/s40037-019-0497-2

**Published:** 2019-02-05

**Authors:** Deepthiman Gowda, Tayla Curran, Apurva Khedagi, Michael Mangold, Faiz Jiwani, Urmi Desai, Rita Charon, Dorene Balmer

**Affiliations:** 1 Department of Medicine, Columbia University Irving Medical Center, Columbia University Vagelos College of Physicians and Surgeons, New York, USA; 2 Columbia University Vagelos College of Physicians and Surgeon, New York, USA; 3 Department of Medicine, Baylor College of Medicine, New York, USA; 4 The Center for Family and Community Medicine, Columbia University Vagelos College of Physicians and Surgeons, New York, USA; 5 Department of Medical Humanities and Ethics, Columbia University Irving Medical Center, Columbia University Vagelos College of Physicians and Surgeons, New York, USA; 6 Department of Pediatrics, Children’s Hospital of Philadelphia, Philadelphia, USA

**Keywords:** Narrative medicine, Health humanities, Interprofessional collaborative practice, Interprofessional education

## Abstract

Interprofessional education (IPE) is a critical component of medical education and is affected by the characteristics of the clinical teams in which students and residents train. However, clinical teams are often shaped by professional silos and hierarchies which may hinder interprofessional collaborative practice (IPCP). Narrative medicine, a branch of health humanities that focuses on close reading, reflective writing, and sharing in groups, could be an innovative approach for improving IPE and IPCP. In this report, we describe the structure, feasibility, and a process-oriented program evaluation of a narrative medicine program implemented in interprofessional team meetings in three academic primary care clinics. Program evaluation revealed that a year-long narrative medicine program with modest monthly exposure was feasible in academic clinical settings. Staff members expressed engagement and acceptability as well as support for ongoing implementation. Program success required administrative buy-in and sustainability may require staff training in narrative medicine.

**Electronic supplementary material:**

The online version of this article (10.1007/s40037-019-0497-2) contains supplementary material, which is available to authorized users.

## Introduction

Interprofessional education (IPE) is a critical component of medical education that prepares trainees to work effectively in healthcare teams. Academic primary care clinics are sites where robust interprofessional coordination is needed to deliver high quality care; they are also settings where much fundamental medical student and resident education takes place [1]. However, clinical teams might not demonstrate successful interprofessional collaborative practice (IPCP) by virtue of not employing the habits of communication and teamwork that are taught to trainees [2]. These conditions are partly due to social and cultural divisions between professions that hinder IPCP and the potentially harmful hierarchies of power that compound these divisions [3–5]. The resultant lack of formative interprofessional modelling for interprofessional students and residents has resulted in calls to enhance teamwork training in workplace settings [2, 6].

Narrative approaches to clinical care, with their focus on allowing space for the diverse voices on interprofessional teams, have the potential to improve interprofessional education and collaborative practice (IPECP) [7]. Narrative medicine, a branch of health humanities that provides training to practice medicine with ‘narrative skills of recognizing, absorbing, interpreting, and being moved to action by the stories of illness’ can be an innovative approach for improving IPECP [8]. Narrative medicine is a discipline that deploys knowledge and methods from humanities and the arts to improve the effectiveness of healthcare. By probing the meanings of oral, written, or visual narratives, narrative medicine equips learners with interpretive and interpersonal skills to recognize the tellers of stories. The methods used include working with groups to: 1) engage with creative works to develop skills of attention, 2) write reflectively to discover and explore personal narratives, and 3) share writing with others to strengthen relationships [8]. Narrative medicine programs have been successfully implemented in educational settings [9]; however, there are few reports of such programs in clinical settings. Furthermore, these programs have been either relatively short-term in duration, not interprofessional, or not situated in primary care settings [10, 11].

We implemented a narrative medicine program in academic primary care clinics with the long-term goals of improving interprofessional communication and relationships. In this report, we focus on: 1) description of the program structure, 2) barriers and catalysts to program implementation, and 3) process-oriented program evaluation.

## Methods

### Program setting and participants

We implemented a 1-year (April 2016 to March 2017) narrative medicine program in three New York Presbyterian Hospital academic primary care clinics that serve as training sites for medical students and resident physicians in family medicine, internal medicine and paediatrics.

We met with clinic leaders before implementation to discuss how narrative medicine might improve teamwork; given shared interest in team development, leaders allotted 30 minutes during monthly required interprofessional team meetings for narrative medicine sessions. In this paper, ‘staff’ refers to attending physicians, resident physicians, nurses, medical assistants, social workers, administrators, and many other clinic personnel. Staff were required to attend the narrative medicine sessions but could opt out of program evaluation. The program evaluation was approved by the Institutional Review Board at the Columbia University Irving Medical Center (protocol # AAAP1351). The work was carried out in accordance with the Declaration of Helsinki including, but not limited to, there being no potential harm to participants, that the anonymity of participants was guaranteed, and that informed consent of participants was obtained.

### Session description

Each 30-minute session consisted of four stages: engaging with a creative work, writing to a prompt, sharing writing in pairs, and sharing writing in a large group [12]. Table 1 details the activities and functions of each stage. Co-author DG facilitated the majority of the sessions. Facilitators of narrative medicine sessions require skills in textual interpretation, group facilitation and application of specific methodology; all program facilitators had masters-level training in narrative medicine. We maintained a consistent structure for each session, but adjusted timing to meet clinical needs. We also adapted creative works used based on expressed staff interest (e. g. a Spanish-language song used as a creative work in clinics staffed by native Spanish speakers).

**Table 1:** The four stages of the narrative medicine session

Session stage (approximate duration)	Description	Specific examples from observation notes	Function	Facilitator’s role
Stage 1: Engaging with a creative work (7 min)	The facilitator leads a discussion of a creative text. The text can be any creative medium including poetry, prose, visual art, music, spoken word, graphic novels, etc.	The painting, ‘The Harvesters’ by Pieter Bruegel (1565), was displayed on a large monitor. The session started with the invitation to observe: ‘Please take a minute to observe the painting in silence.’ During the group discussion of the work, staff noticed the work of harvesting crops in the middle ground and the sharing of a meal in the foreground. They shared their ideas of the likely time and place of the scene. They talked about the tension or balance between work and rest, community and self, men and women. They discussed the idea of teamwork and how the painting related to their own work in the clinic.	The function of this section of the workshop is to engage in close looking. If a written text is used, the activity is of close reading. Through the act of close looking or reading, we aim to strengthen participants’ skills of attention to their world and to themselves.	The facilitator aims to create an open and supportive environment that encourages an exploration of many perspectives. In an attempt to encourage close observation and skills of description, the facilitator asks staff members to find evidence for interpretations offered. For instance, in response to a comment such as ‘The workers in the painting look tired.’ the facilitator might ask, ‘What do you see in the image that makes you say that?’
Stage 2: Writing to a prompt (3–5 min)	The writing prompt is crafted ahead of time and draws from an element (a word, image, metaphor, theme, etc.) used in the creative text.	‘Now we’re going to do some brief writing. Don’t worry about how good a writer you think you are. Just go where the pen takes you. We are mainly interested in what comes up for you in the process of writing. Please write for 5 min to the prompt: *write about the seeds that you are planting*. I will let you know when you have a minute left.’	Prompts are crafted in order to encourage reflective writing about one’s own life. Brief and unplanned writing allows spontaneous expression that explores associations, memories, and emotions that is not analytical or essay writing.	During this stage, the facilitator seeks to create a safe environment of openness, acceptance, and discovery. The facilitator seeks to lessen anxiety that often occurs in such situations of creativity and encourages participants to take risks with one another. Particularly for groups that are not familiar with narrative medicine sessions, the facilitator explicitly invites participants to write with the knowledge that they will share their writing with one another.
Stage 3: Sharing writings in pairs (7–10 min)	Staff are asked to form a pair with someone from a different profession and read their writings to one another.	‘Now pair up with someone who is not in your profession and read your writing to one another. You may need to get up and move to a different part of the room. Listen closely to the reading and pay attention to both *what* the story is telling and *how* it is being told. After you listen, tell your partner what you heard.’ Staff sat closely in pairs so as to hear their partner over a room full of people reading to one another. Leaning in, good eye contact, and nodding seemed to convey careful, attentive listening.	This activity allows for courageous telling and attentive listening. Sharing of one’s writing is an act of risk taking, and when the listener is respectful and attentive there is an opportunity for the building of trust.	The facilitator asks persons to read the text they have written to one another, and not just talk about what they have written. This is intended to allow attention to the text that was produced by the participants. During the reading, the facilitator quietly observes the pairs to make sure all are participating but does not interrupt the sharing. At half-way point, the facilitator reminds the pairs to switch.
Stage 4: Sharing writing in large group (7–10 min)	The large group is reconstituted and the facilitator asks for 2–3 volunteers to share their writing with the group.	‘Ok. Let’s come back to the big group. I want to invite a couple of you to read your writing with the group. Who would like to get us started?’ After each telling, the listeners commented on what they heard.	The content of what is shared is often sensitive in nature, exploring topics like one’s hopes, fears, and personal situation that sometimes results in the expression of emotion. This allows persons in the room to gain new understandings of their colleagues, and to more fully witness their humanity.	The facilitator asks for volunteers to read their text to the whole group. The facilitator allows silence after such an invitation in order to allow participants the time that might be needed to gather up the courage to volunteer to read. The facilitator encourages close listening for form and content of what was written while also listening very closely to what is read. At the close of the session, facilitator may identify themes that came up during the session. In closing, the facilitator might thank the group for their close listening and for trusting of one another with their stories.

### Program evaluation

Evaluation data were derived from: 1) observation notes from narrative medicine sessions by either TC, FJ, or AK; 2) longitudinal semi-structured individual interviews with interprofessional staff from each clinical site at program start, midpoint, and end conducted by either DB, UD or MM (to allow open and critical dialogue, session facilitator DG did not conduct interviews); and 3) 12-question questionnaire exploring barriers and catalysts to participation, and staff’s overall reactions to the program.

### Qualitative data analysis

We used a general inductive approach for qualitative analysis, which asks, ‘What are the core meanings evident in the text, relevant to evaluation objectives?’ [13–15]. DG and AK led the data analysis. They partnered with the team to create codes (words acting as labels for core concepts). They applied codes to data from interview transcripts and observation notes, and managed data in Dedoose.com. At weekly team meetings, DG and AK reviewed the codes to establish links between the data and evaluation objectives. Emergent findings informed iterative interview guide development and real-time program improvement. Given the evaluation focus and the rich and varied sources of data, the team deemed the data sufficient to address the evaluation objectives. Quantitative data from the questionnaire were descriptively analyzed.

## Results

Over a 1-year period, 36 narrative medicine sessions took place at three clinics. We analyzed 32 observation notes and 33 interview transcripts. Fifty out of the 104 unique staff members who attended the sessions during the year completed an end-of-program questionnaire. The questionnaire respondents across sites included 14% attending physicians, 16% resident physicians (representing all 3 years of training), 26% nurses and nurse practitioners, 16% medical assistants, and 28% other staff. Medical students were not involved in program evaluation. Age was reported by 45 respondents; 47% of respondents were less than 40-years-old and 53% were 40-years-old and over. Average duration of employment was 7.3 years (median 6 years and range 4 months to 23 years).

Observation notes and attendance sheets indicated that sessions were attended by an average of 11 staff (82% women and 18% men). Session composition was 21% attending physicians, 9% resident physicians (representing all 3 years of training), 11% nurses and nurse practitioners, 12% medical assistants, and 47% other staff. One or two medical students on their primary care clerkship participated at some sessions at the family medicine site. Fifty-six percent (28/50) of staff surveyed at program conclusion reported they had attended over half the offered sessions.

### Engagement with narrative methods

We observed that staff participated actively during each of the four stages of the narrative medicine sessions. While exploring creative works (Stage 1), staff actively commented on works and listened and responded to others’ observations. Staff remained open to differing viewpoints on creative works. When asked to write to a prompt (Stage 2), staff wrote attentively and without hesitation. Many continued writing, even after a call was made to stop and reconstitute the larger group. When sharing writing in pairs (Stage 3), staff members sat closely and leaned in to listen to one another. We commonly observed laughter and sometimes witnessed expressions of sadness during sharing of writing. During Stage 4, staff routinely volunteered to read their writing to the group, and other staff listened closely and responded to the reading. Staff sometimes stayed after the conclusion of the session to continue conversations.

### Acceptance of program

Based on questionnaires, 94% of staff (47/50) indicated that they would recommend the narrative medicine program to other clinics and 74% (37/50) of staff reported they would continue participating in the program even if it were optional and offered during their free time (before/after clinic or during lunch).

One program participant recalled that at program start, some were hesitant to ‘share personal information,’ particularly with staff from different professions. Despite this initial hesitation, staff actively engaged in the sessions and explored personal stories that often invoked strong emotional responses from both reader and listener (e. g. laughter, tears). This occurred during both pair-sharing and group-sharing; as the year-long program progressed, sharing of personal stories sometimes occurred even earlier in the session during discussion of the creative work.

We observed that the participation in discussion and sharing during sessions was distributed evenly across professions and hierarchy. Several staff members stated that one of the most striking aspects of the program was the communication that opened up across professions and levels of hierarchy that had not occurred with other prior team-building activities at the clinic. Indeed, 40% (20/50) of respondents rated the program’s ability to facilitate interprofessional dialogue as excellent, 26% (13/50) rated it as very good, and 24% (12/50) rated it as good. One staff member said, ‘The best thing I think about narrative medicine … is that it really involved everyone.’ In an interview, a staff member noted that the conversations that occurred at the sessions allowed staff to ‘take our hats off’ and ‘really get to know one another on a different level’.

### Barriers and catalysts to participation

Staff most frequently expressed time constraints and the pull of clinical duties as barriers to program success. A staff member said, ‘I think the activity is great. It’s just, where do we carve out the time that doesn’t affect access for patients?’ Thus, buy-in and support from clinic leaders appeared crucial to program success. Support took the form of protecting time for the meetings, arranging coverage for clinical duties, and clinical leaders’ attendance at sessions. At one clinic, leaders even delayed clinic start time by 30 min with a corresponding reduction in patient load to accommodate the sessions.

## Discussion and implications

This process-oriented program evaluation revealed that a year-long narrative medicine program with modest monthly exposure was successfully implemented in academic clinical settings, as evidenced by staff member engagement and acceptability. Staff members expressed positive regard for the program and supported its ongoing implementation.

Although attendance was required, staff were engaged during sessions and quickly took part in activities. Narrative medicine methods are designed to encourage exploration of one’s personal and professional life. Thus, emotions such as joy and sadness were explored and openly expressed during sessions, in keeping with a recognition that the expression of emotions may be important for clinical care and IPCP [16, 17]. Such expressions occurring earlier in sessions as the program progressed suggests increasing comfort with narrative medicine methods, increasing levels of trust with one another, or both. A prior narrative medicine report argues that safety in groups improves trust and creativity [18]. Indeed, psychological safety in medical and educational contexts may facilitate core behaviours like asking for help or reporting mistakes [19].

In interviews, staff felt that personal connections made across professions and levels of hierarchy were particularly striking. One staff member said that the sessions allowed ‘the whole care team’ to ‘just talk to each other like human beings.’ This points to the potential of art and literature to serve as a democratizing space where persons can gather to explore life experiences as relative equals [7]. Indeed, this effect of narrative medicine may have great value in improving collaborative practice.

We report a narrative medicine program that serves as a feasible model for implementing health humanities in clinical settings. The program’s feasibility was reliant on support from clinic leaders and the availability of a facilitator trained in narrative medicine. Sustainability was not an objective of the project, but it is notable that only the paediatrics clinic, led by a physician with narrative medicine experience, continued monthly narrative medicine activities for an additional year. Staff in that clinic expressed interest in further narrative medicine training. Future narrative medicine interventions will attend to staff training to achieve sustainability. Short, intensive facilitator training programs are under development by the program team to maintain quality while allowing wider dissemination.

Narrative medicine has the potential to improve IPCP by strengthening interprofessional communication and relationships while softening clinical hierarchies; this, in turn, could enhance IPE through positive modelling of interprofessional teamwork. With feasibility and acceptability established in this report, the team’s next steps will focus on more rigorous evaluation of narrative medicine’s impact on outcomes relevant to IPCP such as communication, hierarchy, cohesion, and clinically situated behaviours, in addition to the impact on trainees’ behaviours and attitudes. A limitation of this report is that the number and duration of sessions needed to achieve and sustain benefits of narrative medicine programs remains unknown; this will also be investigated in future work. Finally, research in narrative medicine will increasingly strive to measure patient-oriented outcomes such as patient satisfaction, clinical processes, and clinical outcomes.

### Caption Electronic Supplementary Material

12 Session Narrative Medicine Program: Texts, Writing Prompts and Resources

## Appendix A

### Example of an iteratively developed—semi-structured interview guide

1. You have had Narrative Medicine Sessions in your clinic now for a couple of months. How have those sessions gone so far?
  - What did you think when the idea first was introduced?
  - Did you have any concerns at that time?
  - Has that changed at all?
2. Could you tell me about a time when you felt really engaged in NM work?
  - Who was there?
  - What was it about that particular artwork or writing that was important to you?
3. What’s it been like to write during the sessions?
4. You are asked to share your writing with a partner—what was that like for you?
5. Do you feel that you have learned anything new through the NM exercises?
6. Have you learned something new about your co-workers through the NM sessions?
7. Have you noticed any other changes that have taken place in the clinic since the introduction of the NM sessions? Or any changes you have made?
8. And what are your thoughts about this program that will go on over the coming year? [*these may be better questions for clinical leadership*]
  - Do you have any concerns about it?
  - Do you see any barriers to being able to sustain this?
  - How do you think this might help?
9. The next few questions ask about barriers and facilitators of NM sessions here at X clinic. Sometimes it’s helpful to think about these in 4 broad categories: structural, human resources/people, politics and culture.
  - Lets start with structural.
    I. Is there anything about the structure and function of the clinic that is a barrier?
    II. What about the structure and function of the clinic that might facilitate the NM sessions?
  - Now let’s think about human resource/people—
    I. Barrier
    II. Facilitator
  - Politics
    I. Barrier
    II. Facilitator
  - Culture of the clinic
    I. Barrier
    II. Facilitator

## Appendix B

### Macy project program evaluation: exit survey

**Q1 How many sessions did you attend?**
- □ None
- □ 1–2
- □ 3–5
- □ 6–8
- □ 9–12

**Q2 If you attended less than 12 sessions, please indicate the primary reason(s) for absence.**
- □ Clinical duties
- □ Vacation
- □ Shift starts later
- □ Other (please specify) ____

**Q3 Please rank the following types of text by preference (1 = most preferred, 5 = least preferred)**
- □ Poetry
- □ Painting
- □ Spoken Word
- □ Graphic Novel
- □ Meditation

**Q4 Please indicate the type of text with which you have some experience (check all that apply).**
- □ Writing Poetry
- □ Painting or drawing
- □ Doing spoken word
- □ Creating graphic novels
- □ Meditating
- □ Other (please specify) ____

**Q5 How would you rate the ability of the program leaders to facilitate inter-professional dialogue?**
- □ Poor
- □ Fair
- □ Good
- □ Very Good
- □ Excellent

**Q6 How would you rate the time frame for the sessions?**
- □ Poor
- □ Fair
- □ Good
- □ Very Good
- □ Excellent

**Q7 How would you rate the location for the sessions?**
- □ Poor
- □ Fair
- □ Good
- □ Very Good
- □ Excellent

**Q8 Would you participate in “round 2” if sessions were before or after work?**
- □ Yes
- □ No

Q9 What 2 words would you use to describe the narrative medicine program?

**Q10 Would you recommend this program to other clinics?**
- □ Yes
- □ No

Q11 If you could change one thing about the program (e.g., length or frequency of sessions; location; attendance policy) what would it be?

### Demographics Sheet

Age (please circle)   18–29   30–39   40–49   50–59   60–69 70+

How many years have you worked at this clinic?____

What is your role in the clinic?____
