# Supplementary material for: Implementing an interprofessional narrative medicine program in academic clinics: Feasibility and program evaluation
Source: Perspect Med Educ. 2019 Feb 5;8(1):52–9. doi: 10.1007/s40037-019-0497-2 (PMC6382622; doi:10.1007/s40037-019-0497-2)
Supplement: Supplementary file 1 — 12 Session Narrative Medicine Program: Texts, Writing Prompts and Resources [file 40037_2019_497_MOESM1_ESM.docx]

Table 2. 12 Session Narrative Medicine Program: Texts, Writing Prompts and Resources

| Session # | Type of Text | Text Used | Writing Prompt | * Resources Needed |
| --- | --- | --- | --- | --- |
| 1 | Painting | *The Doctor* by Luke Fildes | “Write about a time when you cared for another.” | Video monitor or AV projector and laptop computer |
| 2 | Spoken Word Video | *Tamara’s Opus* by Joshua Bennett | “Write about what you are listening for.” | Video monitor or AV projector and laptop computer |
| 3 | Video | *Empathy* produced by Cleveland Clinic | Think of a patient you are concerned about. Imagine that patient sitting in your clinic waiting for an appointment. Imagine and write their thoughts using first-person point of view.” | Video monitor or AV projector and laptop computer |
| 4 | Poetry | “The Ship Pounding” by Donald Hall | “Write about a time when the ship left port.” | Photocopies of poem |
| 5 | Poetry | “Kindness” by Naomi Shihab Nye | “Write about a moment of kindness” | Photocopies of poem |
| 6 | Graphic Art | *Mom’s Cancer* by Brian Fies | “Draw a scene of care.” | Color photocopies of pages from graphic novel |
| 7 | Photograph | *Denali* by Deepthiman Gowda | “Write about the journey you are on.” | Video monitor or AV projector and laptop computer |
| 8 | Poetry | “Days”by Philip Larkin | “Describe the days that brought you here.” | Photocopies of poem |
| 9 | Mindfulness | Thich Nhat Hanh YouTube video  “Compassion and Interbeing” and “Brief Meditation: Arriving in Mindful Presence.” www.tarabrach.com | No prompt. The group participated in a mindfulness activity of observing one’s breath and engaged in discussion. | None |
| 10 | Music | “Vivir Mi Vida” by Marc Anthony | “Write about a time of living in the moment.” | Powered audio speaker and smart phone |
| 11 | Painting | *The Harvesters* by Pieter Bruegel | “Write about the seeds you are planting.” | Video monitor or AV projector and laptop computer |
| 12 | Poetry | “The Weighing of the Heart” by Maura Dooley | “Write about a memory of consolation.” | Photocopies of poem |

* The sessions at all sites took place around a central table in conference rooms typically used for clinical or educational discussions. The conference rooms had the capacity to seat approximately 15-20 persons.
